# Correlated MR spectroscopic imaging of breast cancer to investigate metabolites and lipids: acceleration and compressed sensing reconstruction

**DOI:** 10.1259/bjro.20220009

**Published:** 2022-10-12

**Authors:** Ajin Joy, Andres Saucedo, Melissa Joines, Stephanie Lee-Felker, Sumit Kumar, Manoj K Sarma, James Sayre, Maggie DiNome, M. Albert Thomas

**Affiliations:** ^1^ Radiological Sciences, David Geffen School of Medicine, University of California Los Angeles, Los Angeles, CA, United States; ^2^ Physics and Biology in Medicine-Interdepartmental Graduate Program, University of California Los Angeles, Los Angeles, CA, United States; ^3^ Surgery, David Geffen School of Medicine, University of California Los Angeles, Los Angeles, CA, United States

## Abstract

**Objectives:**

The main objective of this work was to detect novel biomarkers in breast cancer by spreading the MR spectra over two dimensions in multiple spatial locations using an accelerated 5D EP-COSI technology.

**Methods:**

The 5D EP-COSI data were non-uniformly undersampled with an acceleration factor of 8 and reconstructed using group sparsity-based compressed sensing reconstruction. Different metabolite and lipid ratios were then quantified and statistically analyzed for significance. Linear discriminant models based on the quantified metabolite and lipid ratios were generated. Spectroscopic images of the quantified metabolite and lipid ratios were also reconstructed.

**Results:**

The 2D COSY spectra generated using the 5D EP-COSI technique showed differences among healthy, benign, and malignant tissues in terms of their mean values of metabolite and lipid ratios, especially the ratios of potential novel biomarkers based on unsaturated fatty acids, myo-inositol, and glycine. It is further shown the potential of choline and unsaturated lipid ratio maps, generated from the quantified COSY signals across multiple locations in the breast, to serve as complementary markers of malignancy that can be added to the multiparametric MR protocol. Discriminant models using metabolite and lipid ratios were found to be statistically significant for classifying benign and malignant tumor from healthy tissues.

**Conclusions:**

Accelerated 5D EP-COSI technique demonstrates the potential to detect novel biomarkers such as glycine, myo-inositol, and unsaturated fatty acids in addition to commonly reported choline in breast cancer, and facilitates metabolite and lipid ratio maps which have the potential to play a significant role in breast cancer detection.

**Advances in knowledge:**

This study presents the first evaluation of a multidimensional MR spectroscopic imaging technique for the detection of potentially novel biomarkers based on glycine, myo-inositol, and unsaturated fatty acids, in addition to commonly reported choline. Spatial mapping of choline and unsaturated fatty acid ratios with respect to water in malignant and benign breast masses are also shown. These metabolic characteristics may serve as additional biomarkers for improving the diagnostic and therapeutic evaluation of breast cancer.

## Introduction

With a lifetime risk of 12.92% and an estimated 281,550 new cases of invasive breast cancer expected to be diagnosed in females in the U.S. alone in 2021,^1^ an early breast cancer diagnosis is important for successful and effective treatment. Detection of breast cancer at an early stage has always been a challenge, as small cancers are usually difficult to discover compared to larger ones, especially in females who have denser breasts. Early detection of malignancy before metastasis outside the breast facilitates improved outcomes and less invasive surgery, and surgical treatment that minimizes deformity has been a key strategy in breast cancer management.^2–11^

The potential role of dynamic contrast-enhanced (DCE) MRI has been reported in the detection and diagnosis of breast cancer.^6–8,11–15^ Increased capillary permeability and an enlarged interstitial space play a dominant role with different enhancements in breast cancer.^16^ In order to record high-resolution breast images, three-dimensional (3D) MRI has been used.^7,8,10–15,17^ The sensitivity of DCE-MRI in detection of malignant breast lesions has been reported in the range of 88–100%.^5,6,18^ The sensitivity of DCE-MRI has recently been reported to be in the range of 92–95% and specificity in the range of 69 to 74%^19^ with another study reporting up to 100% sensitivity and 99% specificity.^20^

Another MR-based functional imaging technique, namely, diffusion-weighted imaging (DWI), probes the microstructure of tissues and is sensitive to the degree to which motion of water molecules is restricted in relation to how packed together cells are.^6,21–28^ Bogner et al demonstrated recently that an optimized DWI imaging protocol at 3.0 Tesla (T) provided a high diagnostic accuracy in 51 patients with only one false-negative lesion and one false-positive lesion.^29^ The sensitivity of DWI in breast cancer has been reported to be in the range of 82–85% and specificity, in the range of 75–82%^30^ while single-voxel spectroscopy has reported an overall sensitivity in the range of 64–82% and specificity in the range of 85–91%.^31^ Multivoxel spectroscopic techniques, on the other hand, have recently reported a sensitivity of 80% and specificity of 74%.^32^ However, further studies using new MR-based technological developments are necessary to assess their role in breast cancer diagnosis and therapeutic evaluation.

MR spectroscopy (MRS) is an efficient biochemical tool for assaying metabolite and lipid concentrations non-invasively in human breast tissues.^33–41^ In addition to the existence of lipids, water, and total choline detected by *in vivo* MRS, *in vitro* MRS in axillary nodes of breast cancer metastases have identified choline (Cho) groups, lactate (Lac), alanine (Ala), and uridine diphosphoglucose (UDPG).^39^ Earlier research on breast cancer using MRS has focused on recording one-dimensional (1D) spectra from single or multiple locations *in vivo* ^33–41^ ; changes in water to fat ratios and Cho levels have been reported in malignant and benign breast masses.^41^ Using localized correlated spectroscopy (L-COSY) in 1 cm^3^ voxels, two-dimensional (2D) spectra were recorded showing increased Cho and reduced lipid ratios.^42–44^ Recording 2D COSY spectra in multiple locations takes several hours of acquisition. However, compressed sensing (CS) allows MR spectroscopic imaging (MRSI) data to be collected in clinically feasible time-frames.^45,46^ Earlier we implemented five-dimensional (5D) echo-planar correlated spectroscopic imaging (EP-COSI), which combined two spectral and three spatial dimensions. Using non-uniform undersampling (NUS) of one spectral and two spatial dimensions, and CS-based reconstruction, 2D COSY spectra were recorded in multiple regions in 3–4 slices, within practical scan time durations.^47^

While single-voxel 1D and 2D MRS can help to analyze the biochemical characteristics of breast tissues, these techniques are generally limited by the small coverage area achievable within a single scan session, and by the relatively higher partial volume effect due to larger voxel size. Multivoxel spectroscopic imaging, on the other hand, covers a larger area of the breast and is usually acquired with a relatively higher spatial resolution due to smaller size of individual voxels compared to single voxel spectroscopy. The 5D EP-COSI technique further increases the coverage area by measuring 2D spectra from multiple voxels within a 3D volume, during a single scan session, and therefore helps to better localize the malignant tissues across the breast.^47^

Several *ex vivo* high-resolution magic angle spinning (HR-MAS) studies of excised breast tissues have reported detection of Cho groups, glycine (Gly), taurine (Tau), myo-inositol (mI), and other metabolites and lipids^40,48,49^ ; detection of these metabolites in addition to Cho *in vivo* is yet to be demonstrated. A recent case report by Bitencourt et al has shown detection of Gly and Cho in one biopsy-proven invasive ductal carcinoma.^50^ Hence, a major goal of this work was to evaluate the recently developed accelerated 5D EP-COSI technology^47^ to detect these novel biomarkers in breast cancer by spreading the spectra over two dimensions.

## Methods and materials

### Subjects

Thirty-one malignant, twenty one benign breast masses, and twenty healthy volunteers were recruited and gave consent according to the on-site institutional review board guidelines. The mean sizes of benign and malignant tumors were 1.93 cm and 2.82 cm, respectively. The difference in mean tumor size between the two groups was found to be not statistically significant (*p* > 0.05) based on a two-sample t-test. The 5D EP-COSI data acquired in five malignant, four benign, and three healthy subjects were excluded in the analysis due to technical failures. Final series in the analysis included subjects with malignant breast masses (*n* = 26, mean age 52 [range:33–71] years; *grade-3* (*n* = 6), *grade-2* (*n* = 11) and *grade-1* (*n* = 9)), benign breast masses (*n* = 17, mean age 37 [range:19–60] years), and healthy controls (*n* = 17, mean age 46 (range:26–64) years). More details about the recruitment can be found in Table 1.

**Table 1.** Characteristics of lesions

Characteristics of lesions	N
***Malignant lesions***	
Number of lesions	31
Mean size (range), cm	3.04 (0.4–6.8)
*Histopathological type*	
Invasive ductal carcinoma (IDC)	17
Invasive lobular carcinoma (ILC)	3
Ductal carcinoma *in situ* (DCIS)	2
IDC+DCIS	6
IDC+ILC	1
ILC+DCIS	1
Other	1
Estrogen Receptor positive	27
Progesterone Receptor positive	25
HER2 positive	5
***Benign lesions***	
Number of lesions	21
Mean size (range), cm	1.93 (0.7–4.5)
*Histopathological type*	
Fibroadenoma	4
Fibro adenomatous proliferation	1
Focal dense stromal fibrosis	1
Fibroepithelial lesion	1
Other	14

### MRI and 5D EP-COSI acquisitions

All scans were acquired on a Siemens 3T Skyra scanner (Siemens Healthineer, Erlangen, Germany). A dedicated “receive” 24-channel phase-array breast coil and a body “transmit” coil was used for all patients and healthy subjects, who were imaged in the prone (head-first) position. *T*_2_-weighted axial and sagittal scans were acquired as references for placing the field-of-view (FOV). Excitation of the spectroscopic volume-of-interest (VOI) for 5D EP-COSI was achieved with an initial 90° RF excitation pulse along the readout direction (x), followed by a pair of adiabatic full passage (AFP) pulses^51^ along the phase-encoding dimension in the anteroposterior direction (y), and a final 90° RF excitation along the second phase-encoded slice dimension (z) with crusher gradients straddling this pulse along all gradient axes for coherence transfer (Supplementary Figure 1A). To encode the indirect spectral dimension (F_1_), the t_1_ increment was placed between the middle 180° AFP pulse and the last 90° pulse. Fat saturation bands were placed around the VOI to minimize the contribution of extraneous lipid signals. Water suppression using a three-pulse sequence^52^ was played before the initial excitation. Signal acquisition started immediately after the last set of crushers.

The 5D EP-COSI data were acquired with FOV of 160×160×120 mm^3^ and matrix size of 16×16×8, resulting in a voxel volume of 1.5 mL. The VOI was localized within the FOV and measured approximately 6×5×4 cm^3^, although this volume varied from subject to subject. The TR/TE were 1500/35 ms, with 64 t_1_ points sampled at 800 µs intervals, corresponding to a spectral bandwidth of 1250 Hz along F_1_ (47). The bipolar echo-planar readout gradient sampled 512 complex t_2_ points with a spectral width of 1190 Hz long F_2_, after separation of even and odd echoes. A non-water suppressed 5D EP-COSI scan with t_1_ = 1 was also acquired for eddy current phase correction.^53^ The total scan time for both the water and non-water suppressed 5D EP-COSI was 28 min, 48 sec. The average full width half maximum (FWHM) of the water peak was 30.4 ± 9.8 Hz over the localized VOI including the cancer and non-cancer locations. In addition, DCE-MRI data was acquired with FOV of 35  ×  35  cm^2^, 176 slices, pixel size  =  0.8 ×  0.8 × 1.1  mm, TR/TE  =  4.10/1.56  ms and flip angle of 10°, prior to injection of 0.1  mmol/kg of gadolinium based contrast agent and five measurements were made after the injection for patients with malignant tumors only.

The T_1_ and T_2_ values of Cho in breast cancer are 1200 ms and 350 ms, and those of water are 1200 ms and 61 ms, respectively.^54^ Hence, it may be noted that the signals may not have fully recovered when using a TR of 1500 ms and T_2_ saturation may be significant at a TE of 35 ms. However, these effects are minimized in the quantitation by reporting the ratios rather than absolute concentrations. While reporting absolute concentrations, these values may be considered for the resonances of Cho and water, while T_1_’s and T_2_’s are unknown for most of the other metabolite and lipid resonances.

### Data reconstruction and post-processing

The 5D EP-COSI data was non-uniformly undersampled with an acceleration factor of 8 using an exponentially-weighted probability distribution applied along the k_y_-k_z_-t_1_ dimensions (masking scheme shown in Supplementary Figure 1B). The undersampled data were reconstructed using a Group Sparsity (GS)-based CS algorithm.^47,55^ CS-based reconstruction techniques assume that the data have a sparse representation in a certain transform domain and undersampling artifacts are removed by maximizing the sparsity of the transform domain coefficients. GS-CS further assumes a structured sparsity in the transform coefficients and operates on adjacent transform coefficients together as groups rather than individually. This approach allows the reconstruction to exploit correlations among the adjacent transform coefficients due to their structured sparsity, and leads to a more accurate reconstruction compared to other conventional l_1_-norm-based CS reconstruction methods.

### Metabolite quantitation

The individual 2D COSY spectra contained contributions from proton resonances along the diagonal (F_1_=F_2_), as well as off-diagonal which are listed in Table 2. The left and right unsaturated fatty acid cross-peaks (UFL and UFR, respectively) and the triglyceryl fat cross-peak (TGF) from here on refer to the average of these cross-peaks located above and below the diagonal.

**Table 2.** Metabolites and lipids identified in the 2D COSY spectra of breast tissue

Spectral Peaks	Locations (F_2_,F_1_) ppm
Methyl Fat (FMETD10)	(0.9, 0.9)
Methylene Fat (FAT14)	(1.4, 1.4)
Methylene Fat (FAT21)	(2.1, 2.1)
Methylene Fat (FAT23)	(2.3, 2.3)
Methylene Fat (FAT29)	(2.9, 2.9)
Choline (CHO32)	(3.25, 3.25)
myo-Inositol+Glycine (MI + GLY)	(3.5, 3.5)
Methylene Glycerol Backbone (MGB42)	(4.2, 4.2)
Water (WAT)	(4.7, 4.7)
Olefinic Fat (UFD54)	(5.4, 5.4)
Unsaturated fatty acid cross-peak, right lower (UFR lower)	(2.4, 5.4)
Unsaturated fatty acid cross-peak, left lower (UFL lower)	(2.9, 5.4)
Triglyceryl fat cross-peak lower, (TGF lower)	(4.3, 5.4)
Unsaturated fatty acid cross-peak, right upper(UFR upper)	(5.4, 2.4)
Unsaturated fatty acid cross-peak, left upper (UFL upper)	(5.4, 2.9)
Triglyceryl fat cross-peak upper (TGF upper)	(5.4, 4.3)

We have quantified the proton 2D peaks in the 5D EP-COSI spectrum using an adaptive peak integration technique that corrects for frequency drifts and confines the integration range for each metabolite on a voxel by voxel basis after eddy current phase correction. After Fourier transforming the non-water suppressed signal along the directly sampled time dimension (S(t_1_, t_2_) to S(t_1_, F_2_)), the dominant lipid peak around 1.4 ppm was zeroed and the resulting spectrum was then inverse Fourier transformed to obtain a water-dominant time signal for the eddy current phase correction. The peak location of each metabolite resonance was identified within the expected frequency range and the magnitude of the peak was integrated above the noise floor within this region. The list of quantified metabolites is shown in Table 2. A prior-knowledge-based quantitation (ProFit) algorithm^56^ and spectrum acquired at higher field strengths can help to quantity additional metabolites like Taurine and other overlapping resonances such as mI and Gly that do not have distinctive vertices inside the expected frequency range. For the purpose of this work, however, we have quantified the combined mI+Gly peaks instead of quantifying them separately, since these peaks are separated by only 0.006 ppm.^50^ When the ratios are computed between 2D and 1D peaks, the voxels are normalized to sum to unity.

### Statistical analysis

Descriptive statistics including means, standard deviations, and 95% confidence intervals were calculated for each metabolite. Student’s t-tests and analysis of variance procedures including Brown-Forsythe and Bonferroni and Games-Howell multiple comparisons were used to determine the predictive values for each detectable metabolite. Fisher’s stepwise linear discriminant analysis was the multivariate procedure used to test significance concerning all combinations of metabolites to find the best linear combination predictive function. A receiver operating characteristic (ROC) analysis was used to assess performance of the function using area under the curve (AUC) as the metric. IBM SPSS Statistics for Windows, Version 24.0. Armonk, NY: IBM Corp., was the software used to perform these analyses. The final statistical analysis of the metabolites and lipids was a non-parametric analysis known as classification and regression tree (CART) analysis, which is a tree-building technique that is unlike traditional data analysis methods in that there are no distributional assumptions. A recursive partitioning of the data was investigated to find the optimum parameters to divide the cohort into malignant and healthy subjects using the CART algorithm (SAS 9.4 (SAS Institute, Cary, NC)).

## Results

### Multivoxel 2D and 1D spectra from 5D EP-COSI

**Figure 1.**
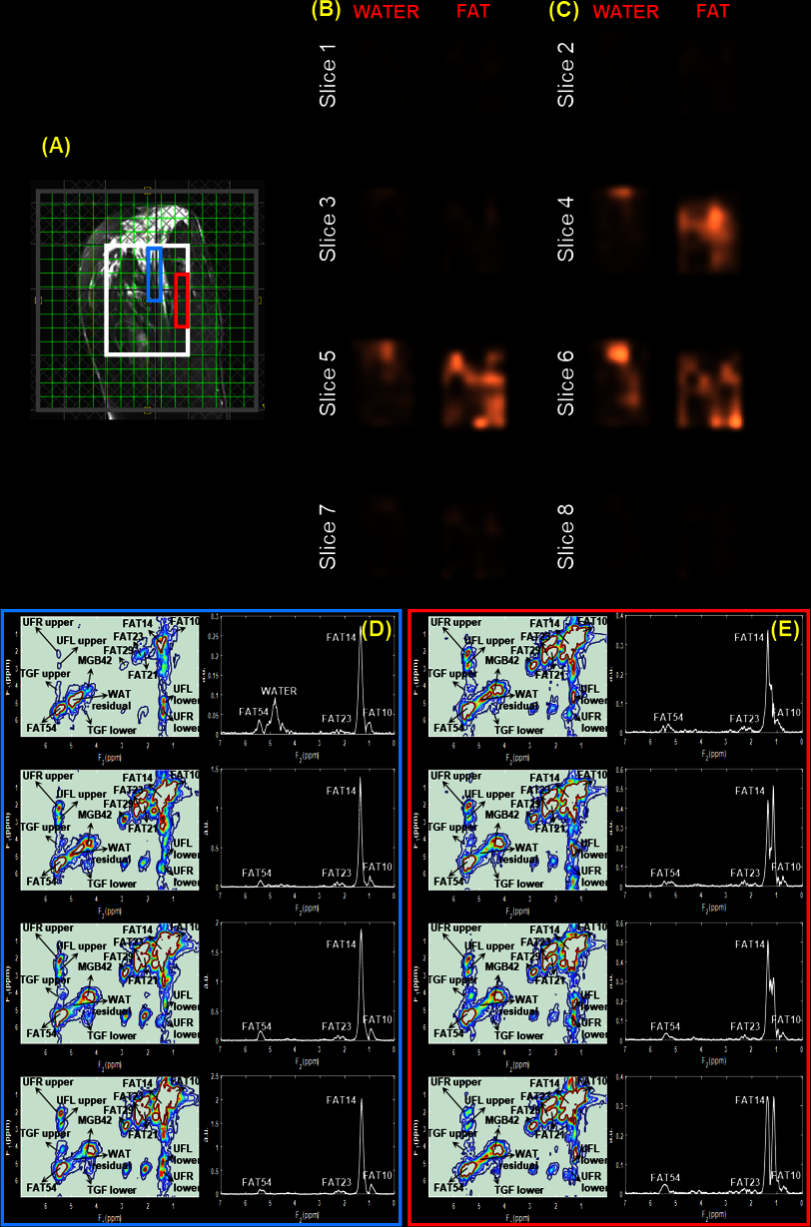
Reconstructed spectra of healthy tissues from a 60-year-old female. (**A**)*T*_2_-weighted MR image with a white box representing the VOI placement. (**B-C**) Metabolite maps of water and fat in eight slices from the non-water suppressed scan. The water and fat maps were reconstructed by projecting each peak into the spatial dimensions. These maps show the spatial variations in the water and fat concentration. (**D-E**) Multivoxel 2D and 1D spectra from two regions within the VOI indicated by blue and red boxes.

**Figure 2.**
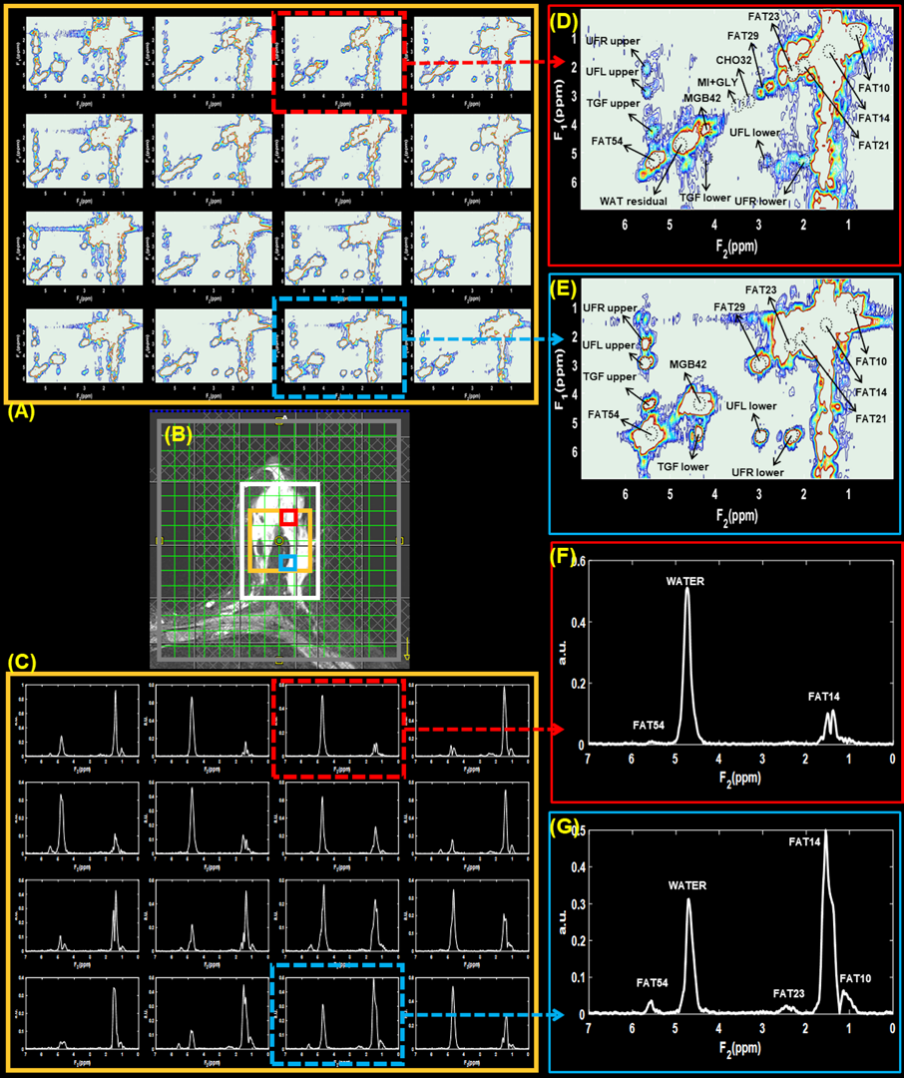
Multivoxel 2D and 1D spectra from a benign lesion (Fibroadenoma, size: 26 mm) in a 32-year-old female. VOI placement on a *T*_2_-weighted MR image with the white box representing VOI is shown in panel (**B**). Panels (**A**) and (**C**) shows 2D water suppressed and 1D non-water suppressed multivoxel spectra from a region within the VOI indicated by the gold-colored box. The voxels highlighted in red and blue boxes point out the spectra from locations containing benign and healthy breast tissues, respectively. 2D and 1D spectra from these locations are shown in panels (**D**)-(**G**).

**Figure 3.**
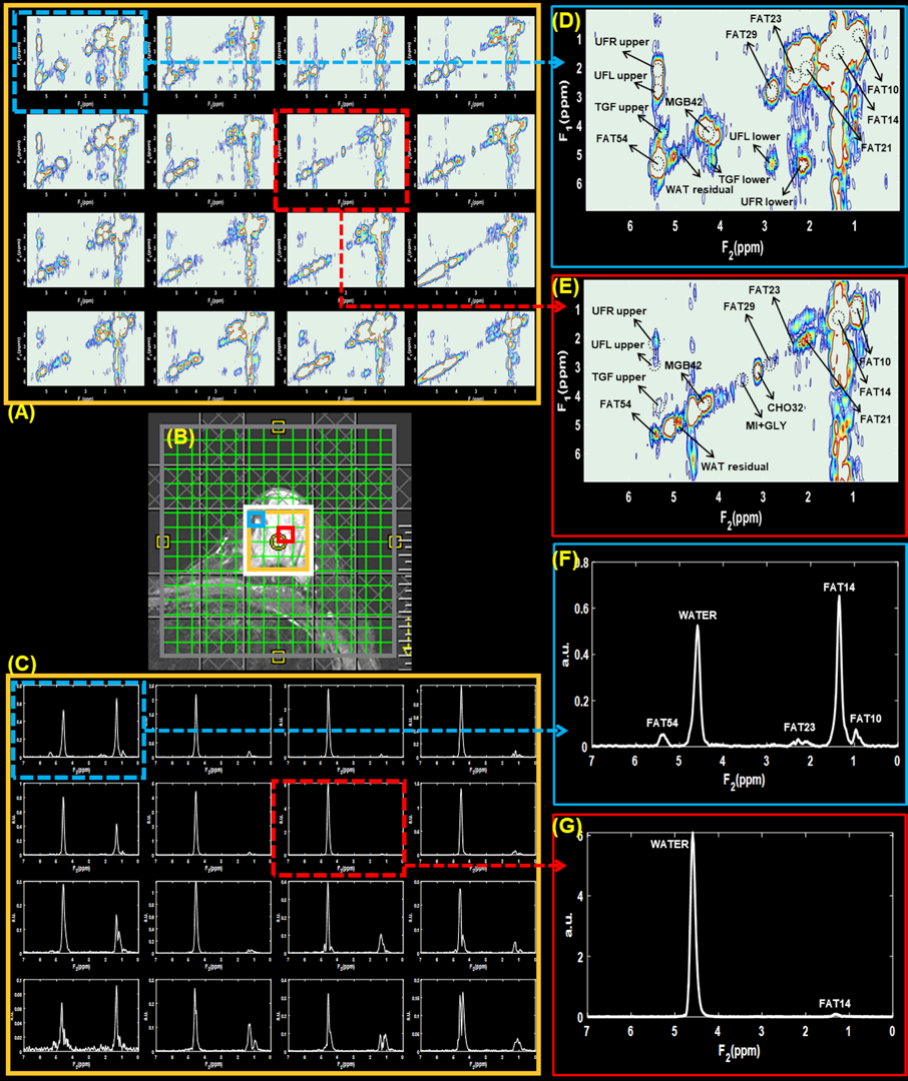
Multivoxel 2D water suppressed and 1D non-water suppressed spectra from lesions identified in 45-year-old malignant patient (Grade three invasive ductal carcinoma and ductal carcinoma *in situ*, estrogen receptor positive, progesterone receptor positive, her2 positive, ki-67 = 20% and BI-RADS 5, size: 33 mm). *T*_2_-weighted MR image with the white box representing the VOI placement is shown in panel (**B**). Panels (**A**) and (**C**) shows 2D water suppressed and 1D non-water suppressed multivoxel spectra from a region within the VOI indicated by the gold-colored box. The voxels highlighted in red and blue boxes point out the spectra from locations containing malignant and healthy breast tissues, respectively. 2D and 1D spectra from these locations are shown in panels (**D**)-(**G**).

**Figure 4.**
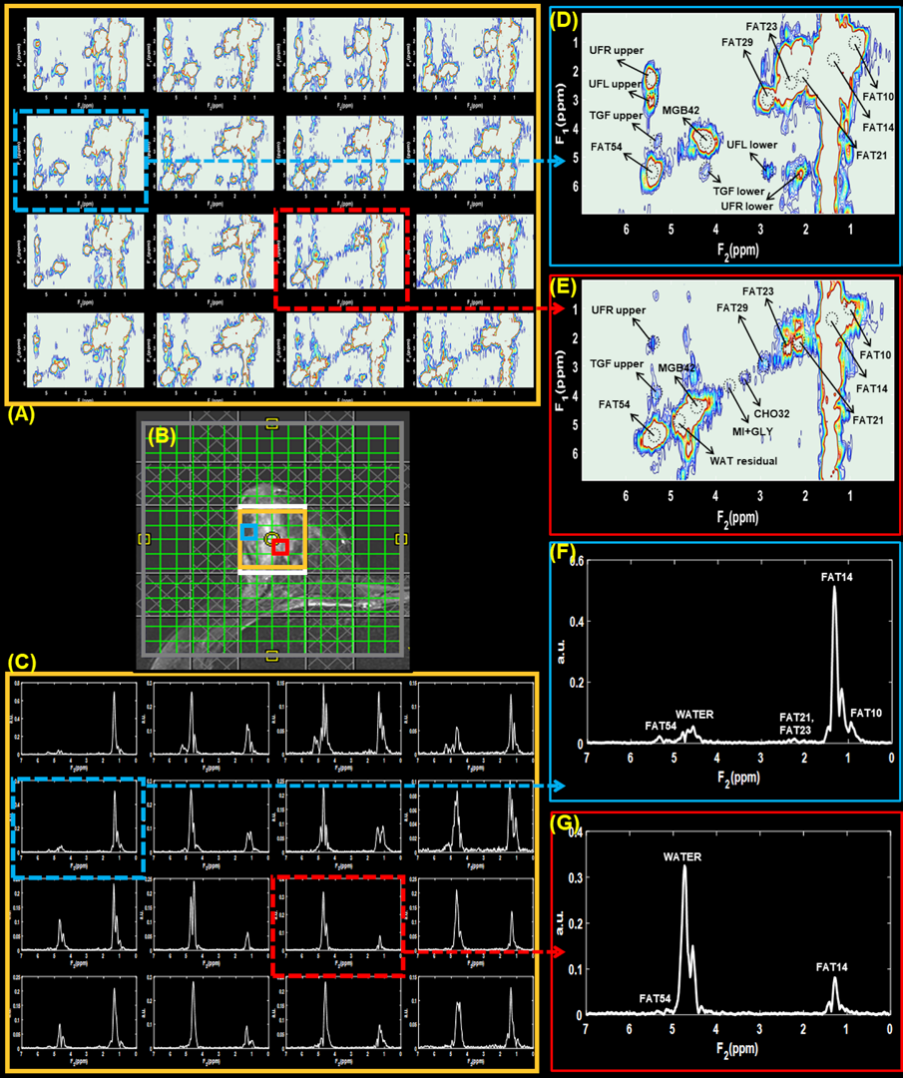
Multivoxel 2D water suppressed and 1D non-water suppressed spectra from lesions identified in 41-year-old malignant patient (Grade three invasive ductal carcinoma and Grade 2 to 3 ductal carcinoma *in situ*, estrogen receptor positive, progesterone receptor positive, her2 positive, ki-67 = 20% and BI-RADS 5, size: 30 mm). *T*_2_-weighted MR image with the white box representing VOI is shown in panel (**B**). Panels (**A**) and (**C**) shows 2D water suppressed and 1D non-water suppressed multivoxel spectra from a region within the VOI indicated by the gold-colored box. The voxels highlighted in red and blue boxes point out the spectra from locations containing malignant and healthy breast tissues, respectively. 2D and 1D spectra from these locations are shown in panels (**D**)-(**G**).

Shown in Figures 1–4 are the MRS VOI placements and the multivoxel 2D water suppressed and 1D non-water suppressed spectra (t_1_ = 1) obtained from healthy, benign, and malignant breast tissues.

Figure 1 shows the reconstructed spectra of healthy tissues from a 60-year-old female. Panel (**A**) illustrates a *T*_2_-weighted MR image with a white box representing the MRS VOI placement. The multivoxel 2D and 1D spectra from two regions within the VOI indicated by blue and red boxes are shown in panels (**D**) and (**E**). Panels (**B**) and (**C**) depict the metabolite maps of water and fat in eight slices from the non-water suppressed scan. The spatially varying intensities in these metabolite maps are representative of the different levels of water and fat concentrations in the glandular and fatty breast tissues. Similar to what is seen in MRI, the water and fat maps were reconstructed by projecting each peak into the spatial dimensions.

Multivoxel 2D and 1D spectra from a benign lesion in a 32-year-old female is shown in Figure 2. Panel (B) shows the MRS VOI placement on a *T*_2_-weighted MR image with the white box representing VOI. The 2D water suppressed and 1D non-water suppressed multivoxel spectra from a region within the VOI (golden box) are shown in panels (A) and (C). The voxels highlighted in red and blue boxes point out the spectra from locations containing benign and healthy breast tissues, respectively. These voxels were extracted and shown in panels (D)-(G) for better clarity. Labels in the spectra show the names of different metabolites and lipids observed.

Multivoxel 2D and 1D spectra selected from malignant lesions identified in 45- and 41-year-old patients are shown in Figures 3 and 4, respectively. The arrangement of panels in both Figures 3 and 4 follows a similar pattern to that of Figure 2, where VOI localization, 2D and 1D spectra shown in the panels (A)-(C), and extracted voxels in panels (D)-(G). The 2D and 1D spectra from voxel locations containing malignant and healthy breast tissues are highlighted in red and blue boxes, respectively.

### Metabolite quantitation and statistical analysis

**Figure 5.**
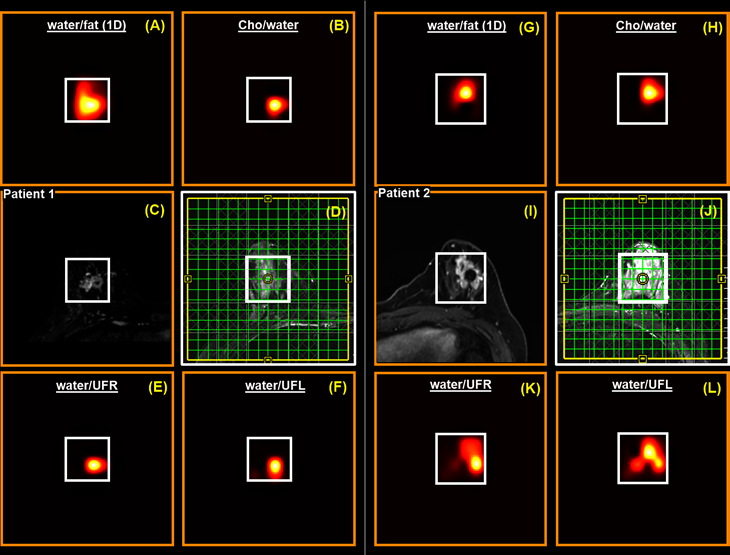
Chemical shift ratio images (spatial distribution of metabolite ratios) corresponding to the multivoxel spectra shown in Figures 3 and 4. Panels (**A**)-(**F**) and (**G**)-(**L**) correspond to 41- and 45-year-old females with malignant breast tissues as classified by radiologists. VOI placement of the two patients are shown in (**D**) and (**J**), and the corresponding contrast-enhanced MRI images are shown in (**C**) and (**I**). Other panels show the spatial distribution of water-to-fat (1D) ratio ((**A**), (**G**)), Choline-to-water ratio ((**B**), (**H**)), water-to-UFR ratio ((**E**), (**K**)) and water-to-UFL ratio ((**F**), (**L**)).

Figure 5 depicts the chemical shift ratio images (spatial distribution of metabolite ratios) of selected metabolites from the multivoxel spectra shown in Figures 3 and 4. Panels (**A**)-(**E**) and (**F**)-(**J**) correspond to 41- and 45-year-old females with malignant breast masses as classified by our radiologists. MRS VOI placement for the two patients are shown in (**C**) and (**H**). Other panels illustrate the spatial distribution of the water-to-fat (1D) ratio ((**A**), (**F**)), choline-to-water ratio ((**B**), (**G**)), water-to-UFR ratio ((**D**), (**I**)) and water-to-UFL ratio ((**E**), (**J**)). Since the ratios of UFL and UFR to water tend to decrease in the presence of malignant tissues, the water-to-UFL and water-to-UFR ratios are shown for better clarity. The bright signal shows that both UFL- and UFR-to-water ratios are lower in the malignant tissues. This trend is in contrast to the Choline-to-water ratio map where the bright signal indicates the region with elevated Choline-to-water ratios.

**Figure 6.**
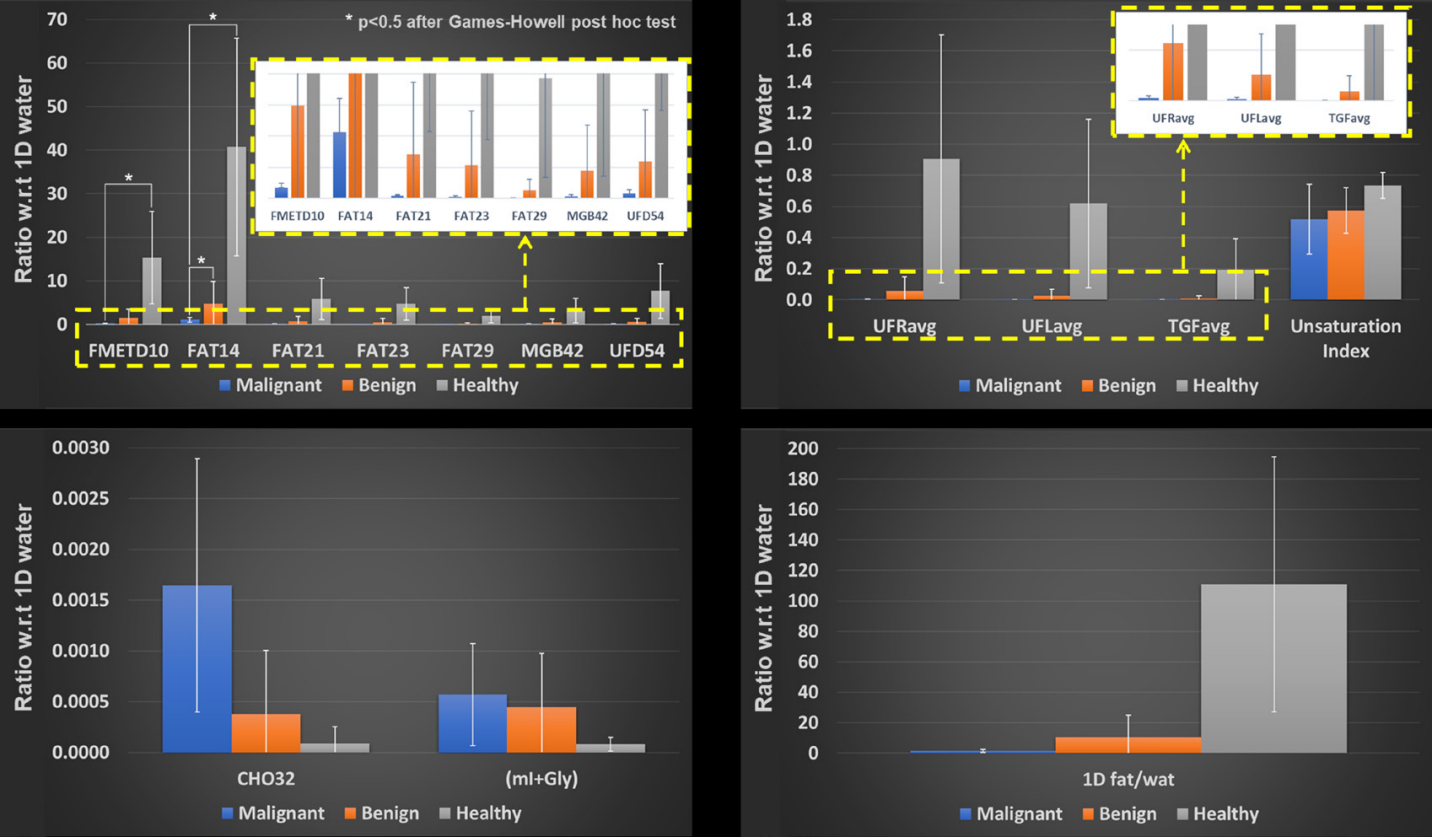
Mean (95% CI) of different metabolite and lipid ratios with respect to 1D water comparing malignant, benign and healthy breasts.

**Figure 7.**
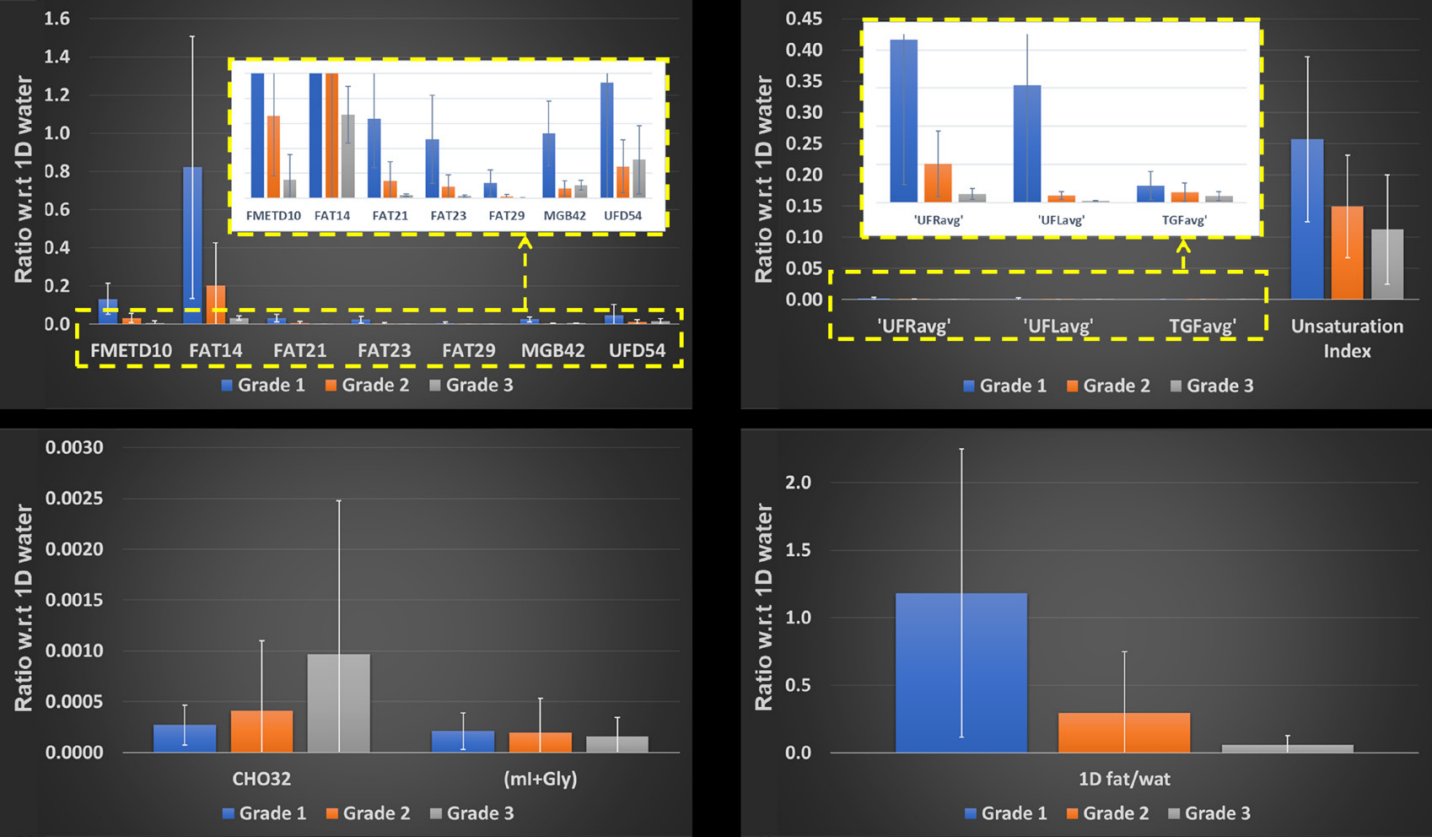
Mean (95% CI) of different metabolite and lipid ratios with respect to 1D water comparing malignant Grade 1, 2 and 3.

The difference in mean tumor size between the malignant and benign groups was found to be not statistically significant (*p* > 0.05) based on a two-sample t-test. Bar graphs showing the means (95% CI) of different ratios of 2D metabolites and lipids with respect to 1D water are presented in Figures 6 and 7. Figure 6 compares the differences in malignant, benign, and healthy subjects. Because of the differences in variances among these three groups, Brown-Forsythe and Welch’s test were conducted. The results from the Brown-Forsythe test indicated a significant difference among the three groups in FMETD10: *p* = 0.010; FAT14: *p* = 0.004; FAT21: *p* = 0.029; FAT23: *p* = 0.025; FAT29: *p* = 0.024; CHO32: *p* = 0.041; MGB42: *p* = 0.048; UFD54: *p* = 0.024; UFRavg: *p* = 0.034 and UFLavg: *p* = 0.031. In addition, the 1D fat to 1D water ratio was also found to be significant with *p* = 0.017. Games-Howell post hoc tests further indicated that FMETD10 and FAT14 differed significantly between healthy and malignant groups with *p* < 0.05, in addition to FAT14 differing significantly between healthy and benign groups with *p* < 0.05. Figure 7 illustrates the difference in malignant Grades 1, 2, and 3. A Brown-Forsythe test for the equality of means, conducted due to differences in variance, did not indicate a statistical significance between different cancer grades.

### Discriminant function and CART analysis

**Figure 8.**
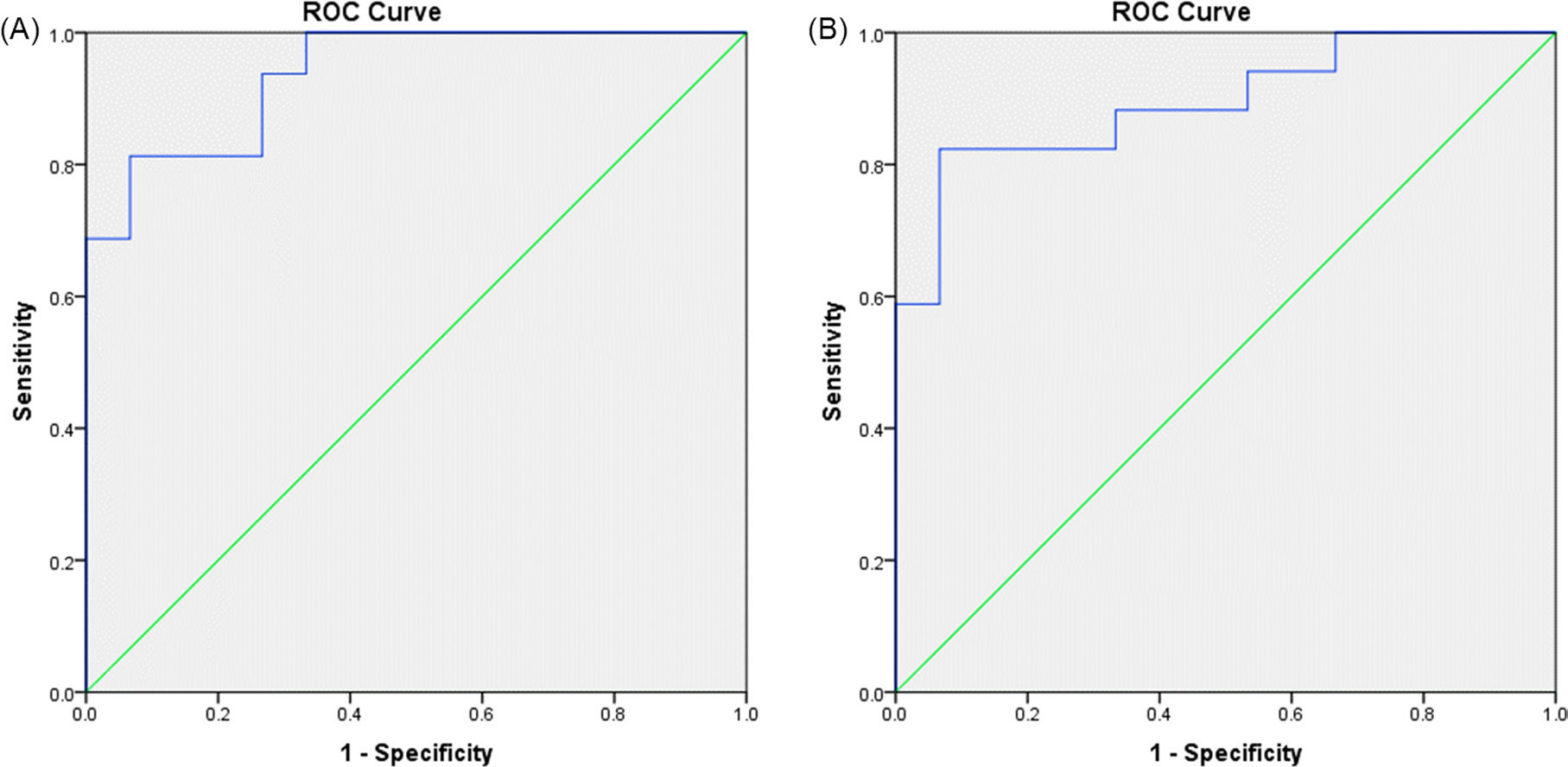
(**A**) ROC curve of the FAT14+CHO32 ratios for differentiating malignant from healthy breast tissues, with an AUC of 0.938 (95% CI: 0.859–1). (**B**) ROC curve of the FAT14 ratio for differentiating benign from healthy breast tissues, with an AUC of 0.894 (95% CI: 0.782–1).

Linear discriminant analysis for healthy and malignant groups based on the ratios of 2D metabolites and lipids to 1D water yielded a statistically significant discriminant model using FAT14 (Wilks’s Λ = .75, *F* ^1,29^ = 9.660, *p* = 0.004) and CHO32 (Wilks’s Λ = .655, *F* ^2,28^ = 7.387, *p* = 0.003) with an AUC of 0.938 (95% CI: 0.859–1). The corresponding ROC curve is shown in Figure 8(A). Ratios of FAT14 and CHO32 to 1D water in the representative voxels containing malignant and healthy tissues from patients and healthy controls were used for this analysis. As reaffirmation of this model, a non-parametric CART analysis was also performed which yielded an AUC of 0.965 with 100% sensitivity and 80% specificity. The discriminant function analysis used leave-one-out validation and the CART analysis used cross-validation. Voxels from control group representing healthy tissues and voxels from patient group containing malignant tissues were used for the analyses. While the discriminant functions identifying benign from malignant masses was not found to be statistically significant, the analysis yielded a significant discriminant model based on FAT14 (Wilks’s Λ = 0.789, *F* ^1,30^ = 8.035, *p* = 0.008) for discriminating healthy and benign groups with an AUC of 0.894 (95% CI: 0.782–1). The corresponding ROC curve is shown in Figure 8(B). The non-parametric CART analysis for this model yielded an AUC of 0.8961 with 94.12% sensitivity and 80% specificity.

## Discussion

In this work, prospectively undersampled 5D EP-COSI data were reconstructed using GS-CS, and 2D COSY spectra from multiple locations in malignant breast masses were analyzed and compared with spectra extracted from benign and healthy breast tissues. In addition to the spectral dispersion along two dimensions, which helps to distinguish and quantify metabolite and lipid markers such as Cho, mI + Gly, UFR and UFL, the multivoxel acquisition technique yields spatial maps of metabolite ratios, which can play a key role in cancer detection (Figure 5).

While variations in water and fat resonances are commonly observed in malignant tissues and have been reported to be useful in identifying malignancy,^41,57^ these variations can become ambiguous in benign tissues and healthy glandular regions, since these also often contain elevated water signal. In this respect, we have shown that the Cho ratio map, generated by quantifying the signal acquired from multiple locations in the breast, can serve as an additional marker of malignancy, complimentary to findings offered by DCE-MRI/DWI.^6,7,11^ Although the water-to-fat ratio tends to be elevated in the malignant tissues, the water signal from the surrounding glandular region can at times introduce a degree of ambiguity in the qualitative comparison. The Cho ratio map generated from the quantified 5D EP-COSI data, on the other hand, can be less ambiguous because the elevated Cho levels are confined to the location of malignant tissues. While the Cho ratio map can play a role in tumor detection similar to DCE-MRI, the lower spatial resolution is currently a limiting factor. The statistically significant linear combination predictive function using polymethelyne fat (1.4ppm) and Cho (3.25ppm) further shows the strong capacity of 5D EP-COSI for detection of malignant tissues.

Although not found to be statistically significant in the current analysis, the findings in this work also point toward the use of other potential bio-markers, such as the unsaturation index, mI, Gly and UFL or UFR spatial distribution maps. UFL-to-water and UFR-to-water ratios decrease in malignant tissues as compared to healthy ones. Hence, the higher intensities in the inverse of the UFL- and UFR-to-water ratio maps (Figure 5) can ideally serve as distinctive markers for malignancy. In practice, however, the close proximity of UFR to t_1_ ridges from the high-intensity methyl and methylene peaks makes UFL more reliable than UFR. This effect is seen in the spectra from the patient two in Figure 5.

Tumor development is reported to be associated with structural changes in lipids that are responsible for a significant reduction in their total unsaturation.^58^ Improved dispersion of resonances in the 2D COSY spectrum allows us to derive additional measures of fatty acid composition, particularly the unsaturation index, which is defined as the ratio of the cross-peak volumes of UFL-to-UFR and is a measure of the degree of lipid unsaturation.^59^ The 5D EP-COSI technique can determine the unsaturation indices in multiple breast regions, which provides an additional tool for characterizing tumor progression.

In addition to measuring lipid-based biomarkers, 5D EP-COSI also allows the *in vivo* detection and quantitation of metabolites like mI and Gly. Previous reports have only shown the role of these metabolites from *ex vivo* breast cancer tissues. It has been reported that tumors larger than 2 cm had significantly higher concentrations of Gly as well as Cho compared to smaller tumors, and this metabolic change has been suggested as a prognostic biomarker since high Gly levels were associated with a poor prognosis.^40^ mI on the other hand has been shown to be involved in hormone signal transduction, formation of glucuronate precursors for detoxification, and also function as an osmoregulator in the different stages of malignant transformation.^60^ Unfortunately, mI and Gly cannot reliably be quantified separately at 3T, even with prior-knowledge based quantitation algorithms. Nevertheless, our analysis has shown marked differences in the peak volumes of mI+Gly among the malignant, benign, and healthy cohorts (Figures 6 and 7), although these differences were not proven to be statistically significant. Further analysis in a larger cohort of subjects may be required to show significant effects of these potential bio-markers.

One of the limitations of this study is due to partial volume effects which can affect the analysis since the size of lesions can be smaller than the voxel resolution used in spectroscopic imaging. Additional improvements in the acquisition and reconstruction technique, such as greater robustness of the sequence to patient motion, are expected to make the lipid-based and mI+Gly biomarkers play a more significant role alongside Cho in cancer detection.

Another limitation of this study is the small subset of data within each grade and subtype of cancer, which prevents the finding of a more definite, statistically significant marker to distinguish the various cancer types and grades. Furthermore, the nature of low-grade DCIS and ILC can affect the sensitivity of MRS detection. DCIS, for example, generally appears as small splashes across various breast regions in MRI, while ILC is initially confined to small lobules. These subtypes could, therefore, become indistinguishable, especially as partial volume effects tend to confound the resonances from cancerous tissues with those from surrounding healthy tissues, due to the low voxel resolution. Furthermore, the resonances due to metabolites such as mI and Gly tend to overlap at 3T, and require prior knowledge-based algorithms to quantify them separately at higher field strengths.

## Conclusion

In this first demonstration of validating the 5D EP-COSI technique to detect biomarkers in malignant and benign breast lesions, we have shown that in addition to metabolite and lipid ratios, metabolite ratio maps generated by quantifying the signal from multiple locations in the breast can serve as markers of malignancy complimentary to those offered by DCE-MRI/DWI. In addition to choline groups, detection of glycine and myo-inositol can facilitate future therapeutic evaluation in breast cancer using these novel biomarkers.^50^ Statistically significant discriminant models based on metabolite and lipid ratios further strengthen the potential of this technique to play a major role in therapeutic evaluation of breast cancer.
